# Intraepithelial CD15 infiltration identifies high-grade anal dysplasia in people with HIV

**DOI:** 10.1172/jci.insight.175251

**Published:** 2024-06-20

**Authors:** Joaquín Burgos, Aleix Benítez-Martínez, Cristina Mancebo, Núria Massana, Antonio Astorga-Gamaza, Josep Castellvi, Stefania Landolfi, Adrià Curran, Jorge N. Garcia-Perez, Vicenç Falcó, María J. Buzón, Meritxell Genescà

**Affiliations:** 1 Infectious Diseases Department, Vall d’Hebron Institut de Recerca (VHIR), Vall d’Hebron Hospital Universitari, Vall d’Hebron Barcelona Hospital Campus, Barcelona, Spain.; 2 Pathology Department, Vall d’Hebron Hospital Universitari, Vall d’Hebron Barcelona Hospital Campus, Barcelona, Spain.; 3 Departament de Ciències Morfològiques, Universitat Autònoma de Barcelona, Bellaterra, Spain.

**Keywords:** Immunology, Infectious disease, Adaptive immunity, Cervical cancer, Innate immunity

## Abstract

Men who have sex with men (MSM) with HIV are at high risk for squamous intraepithelial lesion (SIL) and anal cancer. Identifying local immunological mechanisms involved in the development of anal dysplasia could aid treatment and diagnostics. Here, we studied 111 anal biopsies obtained from 101 MSM with HIV, who participated in an anal screening program. We first assessed multiple immune subsets by flow cytometry, in addition to histological examination, in a discovery cohort. Selected molecules were further evaluated by immunohistochemistry in a validation cohort. Pathological samples were characterized by the presence of resident memory T cells with low expression of CD103 and by changes in natural killer cell subsets, affecting residency and activation. Furthermore, potentially immunosuppressive subsets, including CD15^+^CD16^+^ mature neutrophils, gradually increased as the anal lesion progressed. Immunohistochemistry verified the association between the presence of CD15 in the epithelium and SIL diagnosis for the correlation with high-grade SIL. A complex immunological environment with imbalanced proportions of resident effectors and immune-suppressive subsets characterized pathological samples. Neutrophil infiltration, determined by CD15 staining, may represent a valuable pathological marker associated with the grade of dysplasia.

## Abstract 2

Assessment of changes in relevant anal dysplasia–associated mucosal immune subsets provides potential targets for immunotherapy and a complementary biomarker for current diagnostics.

## Graphical abstract

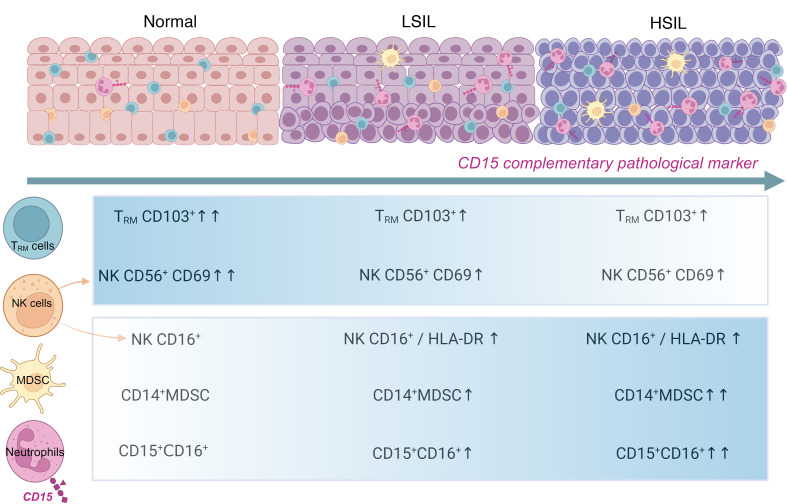

## Introduction

Anal cancer is considered infrequent in the general population (1). However, in selected populations, such as men who have sex with men (MSM) with HIV, anal cancer occurs at rising rates and is currently one of the most common non-AIDS-defining cancers (2). Infection by high-risk human papillomavirus (HR-HPV) at the squamocolumnar transition zone is considered the main etiological agent of anal cancer (3). Persistent HPV infection is able to induce a series of changes in the transitional epithelium that lead to the development of low-grade squamous intraepithelial lesion (LSIL), which can progress to high-grade squamous intraepithelial lesion (HSIL), considered the direct precursor of invasive anal cancer (4, 5).

Anal SILs are histologically identical among people with HIV (PWH) and uninfected individuals; however, they are more prevalent and likely to persist and progress to anal cancer in the first group, even among those in which combination antiretroviral therapy (cART) maintains viral suppression and induces immunological recovery (2, 6). Multiple factors related to the local interaction and potentiation between HIV and HPV may explain this increase in prevalence and associated pathology in PWH, including oncogenic effects and overall impact on local immunity (7–10). Of particular importance may be the persistent depletion of CD4^+^ T cells from the mucosal compartments in PWH who have been treated during chronic infection (11, 12), which may create a more favorable microenvironment for precancerous lesions to develop and progress. In this sense, altered cell-mediated immunity has been associated with increased HPV infection and disease (13), while immune responses orchestrate regression of HPV-related lesions (14).

Screening and treating HSIL have recently been demonstrated to be effective for cancer prevention in PWH (15). However, a reliable biomarker that indicates the risk to develop anal cancer has not yet been identified, and even classifying intermediate lesions of SIL is still challenging (16). Most studies aiming to expand the understanding of anal dysplasia progression and identify potential biomarkers have focused on the genes and/or proteins involved in HPV-mediated carcinogenesis (17, 18). In contrast, studies focusing on the local immune microenvironment surrounding anal lesions are scarce (19), although disturbances in the local microenvironment may play a critical role in the development of anal cancer precursors (20, 21). Thus, phenotyping the immune landscape surrounding dysplastic lesions could provide new insights on the immunopathology of these persistent infections, which in turn may allow the identification of new biomarkers.

In light of the limited data available about the immune microenvironment that differentiate normal epithelium from anal dysplastic lesions and the limitations of diagnostic tools of HSIL, we conducted a study to evaluate immunological subsets in the anal mucosa of MSM with HIV who participated in an anal screening program. The main goal of this study was to characterize the immune environment where lesions develop to identify biomarkers that can contribute to diagnosing HSIL. Based on a pathological diagnostic, we observed divergent trends in resident lymphocyte populations and myeloid-derived suppressor cells and neutrophils. Ultimately, the epithelial infiltration of CD15^+^ neutrophils associated with pathology provides a biomarker of interest for assisting HSIL diagnosis and future immunological interventions.

## Results

### Cohort characteristics.

The discovery cohort comprised 47 cART-treated MSM with HIV, with a total of 54 anal samples. All analyses, including flow cytometry on fresh samples, were conducted simultaneously during screening in this cross-sectional study. Anal samples were subsequently classified based on histological analyses as normal (*n* = 24 samples, including 21 individuals), LSIL (*n* = 24 samples, including 20 individuals), and HSIL (*n* = 6 samples, including 6 individuals). In 7 of these individuals, we had concomitant paired samples, wherein 1 was classified as normal and the other as LSIL. The validation cohort included 54 MSM with HIV, with a total of 57 anal samples classified as normal (*n* = 12 samples, including 12 individuals), LSIL (*n* = 25 samples, including 22 individuals), and HSIL (*n* = 20 samples, obtained from 19 individuals). Of note, 8 of these patients were also included in the discovery cohort, though with different samples (in terms of time point and or localization). Table 1 and Table 2 show a summary of the participant characteristics related to HIV and other relevant parameters from both cohorts.

### Expression of CD103 in resident memory lymphocytes is diminished in pathological samples.

**Figure 1:**
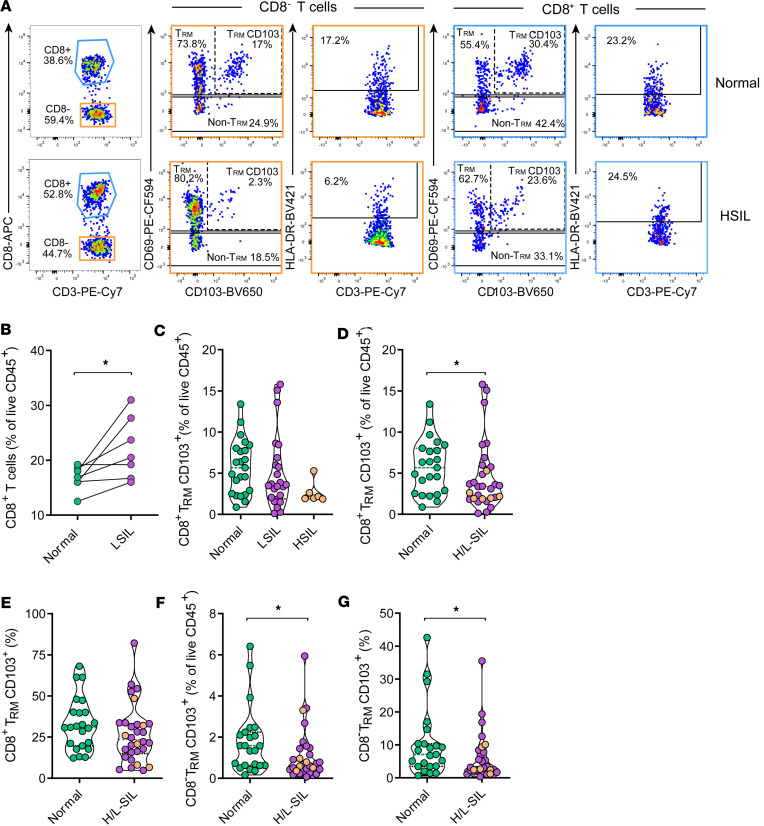
Changes in CD3^+^ T lymphocyte populations associate with SIL. (**A**) Examples of the gating strategies used to identify T cell subsets in a normal anal sample (top) and in a high-grade squamous intraepithelial lesion sample (HSIL, bottom). Sequential gating from left to right was used to identify 4 populations of interest within CD8^–^ T cells (in orange) or CD8^+^ T cells (in blue): T_RM_ (CD69^+^CD103^–^), T_RM_ CD103^+^, non-T_RM_ (CD69^–^CD103^–^), and activated HLA-DR^+^ T cells. (**B**) Frequencies of total CD8^+^ T lymphocytes in paired concomitant normal and LSIL samples from the same individual analyzed by the Wilcoxon signed-rank test. *P < 0.05. (**C** and **D**) Frequencies of CD8^+^ T_RM_ CD103^+^ lymphocytes out of all living CD45^+^ cells in (**C**) the 3 study groups or in (**D**) normal (in green) versus pathological (H/L-SIL, in purple; HSIL are highlighted in brown) samples. (**E**) Frequencies of CD8^+^ T_RM_ CD103^+^ lymphocytes out of total CD8^+^ T lymphocytes in normal versus pathological samples. (**F**) Frequencies of CD8^–^ T_RM_ in normal versus pathological samples. (**G**) Frequencies of CD8^–^ T_RM_ CD103^+^ lymphocytes out of total CD8^–^ T lymphocytes in normal versus pathological samples. Data are represented as a violin plot; horizontal lines are median and interquartile range. Statistical comparisons using nonparametric Kruskal-Wallis test with Dunn’s post hoc test for multiple comparisons and Mann-Whitney *U* test for 2-group analyses are shown: *P < 0.05.

To study immune populations located in the anal biopsies, after selecting live single CD45^+^ cells, we delineated 3 major subsets: T lymphocytes, NK cells, and specific myeloid populations. The flow cytometry gating strategy used for all samples is shown in Supplemental Figure 1; supplemental material available online with this article; https://doi.org/10.1172/jci.insight.175251DS1 The median count of viable hematopoietic CD45^+^ cells retrieved from each biopsy sample is presented in Table 1 and Table 2, which indicates a consistent trend toward increased CD45^+^ infiltration in pathological samples compared with normal ones. Of note, this value was extracted from the acquisition of the entire sample by flow cytometry, without normalization by sample weight or determination of the absolute count after digestion, and thus has limited accuracy. Regarding the analyses of T cells derived from anal biopsies, Figure 1A displays 2 representative samples showing the frequency of CD8^–^ (which were >97% CD4^+^CD3^+^ T cells, Supplemental Figure 2) and CD8^+^CD3^+^ T cell subsets analyzed in normal and HSIL biopsies. In these subsets, we determined lymphocyte activation by HLA-DR expression and tissue residency by CD69 combined with CD103 expression (11). Although the overall frequency of CD8^–^ or CD8^+^ T lymphocytes, out of live CD45^+^ cells, did not vary significantly among the different groups, the analyses of CD8^+^ T cells in paired samples from the same individual, in which both normal and LSIL biopsies were available, revealed a higher total frequency in LSIL samples compared with normal samples (*P* = 0.031, Figure 1B). In contrast, the fraction of CD8^+^ resident memory T cells (T_RM_) expressing CD103^+^ decreased with increasing pathology, showing a trend when comparing normal and HSIL samples (*P* = 0.08, Figure 1C). Indeed, when considering pathological samples as a single group, the trend for CD8^+^ T_RM_ expressing CD103^+^ remained (*P* = 0.063, Figure 1D). However, this difference was lost when displayed as the percentage of total CD8^+^ T cells (Figure 1E). Further, CD8^–^ T_RM_ CD103^+^ cells were significantly lower in pathological samples compared with nonpathological biopsies when analyzed as the percentage of CD45^+^ live cells (*P* = 0.024, Figure 1F) and of CD8^–^ T cells (*P* = 0.036, Figure 1G).

To disentangle the effect of the grade of dysplasia from the effect of having confounding factors such as age, nadir CD4, time on cART, or the presence of HR-HPV on the frequency of T_RM_ subsets, we separated the pathological and nonpathological samples in a post hoc analysis into 2 groups based on these factors (Supplemental Figure 3). Overall, the median frequency of CD8^–^ or CD8^+^ T_RM_ CD103^+^ in normal biopsies was higher than in pathological samples in all comparisons. Further, differences in the frequency of CD8^–^ T_RM_ CD103^+^ between these 2 groups of samples were kept statistically significant for the group with nadir CD4 below 350 cells (Supplemental Figure 3B) and for both subsets when only considering samples without HR-HPV16/18 genotypes (Supplemental Figure 3D). Thus, while CD8^+^ T cell infiltration appeared to be associated with an LSIL diagnostic, the proportion of T_RM_ expressing CD103^+^ was reduced as the level of dysplasia progressed. This reduction was more pronounced for CD8^–^ T_RM_ lymphocytes and was not affected by age, level of nadir CD4, time on cART, or the presence of HR-HPV16/18 genotypes.

### NK cells expressing CD56 are perturbed in pathological samples.

**Figure 2:**
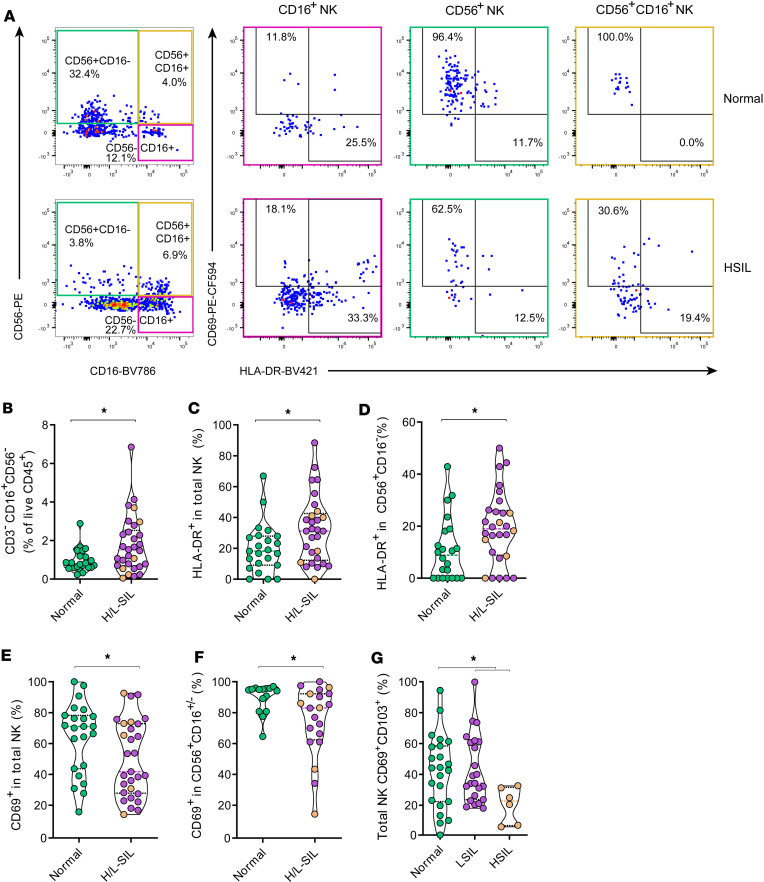
Frequency of NK lymphocyte populations associates with SIL. (**A**) Flow cytometry gating strategy quantifying anal tissue CD3^–^ NK subsets for a nonpathological sample (top) and for a high-grade squamous intraepithelial lesion sample (HSIL, bottom). Expression of CD16^+^CD56^–^ (pink), CD16^–^CD56^+^ (green), and CD16^+^CD56^+^ (yellow) was used to identify NK subsets, in which the activation/resident memory marker (CD69) and the activation marker (HLA-DR) were assessed. (**B**) Frequency of CD3^–^CD16^+^CD56^–^ NK cells out of all living CD45^+^ cells in normal (in green) versus pathological (H/L-SIL, in purple; HSIL are highlighted in brown) samples. (**C**) Frequency of HLA-DR expression in total NK cells in normal versus pathological (H/L-SIL). (**D**) Frequency of HLA-DR expression in CD56^+^CD16^–^ NK cells in normal versus pathological (H/L-SIL). (**E**) Frequency of CD69 expression in total NK cells in normal versus pathological (H/L-SIL). (**F**) Frequency of CD56^+^CD16^+/–^ NK cells expressing CD69 in normal versus pathological tissue samples. (**G**) Frequency of CD69^+^CD103^+^ cells in total NK cells in normal (green), LSIL (purple), and HSIL (brown) anal samples. Data are represented as a violin plot; horizontal lines are median and interquartile range. Statistical comparisons using nonparametric Kruskal-Wallis test with Dunn’s post hoc test for multiple comparisons and Mann-Whitney *U* test for 2-group analyses are shown: *P < 0.05.

We then analyzed the frequency of CD3^–^ lymphocytes based on their expression of CD16 or CD56, as the major NK subsets in tissue samples, as shown in the representative examples (Figure 2A). For these analyses we considered CD16^–^CD56^+^ NK, CD16^+^CD56^+^ NK, and CD16^+^CD56^–^ NK subsets individually and also all together, referred to as total NK cells. Overall, a larger percentage of CD16^+^CD56^–^ NK cells tended to accumulate in pathological samples compared with normal samples (*P* = 0.038; Figure 2B). For each NK cell subset, we also analyzed the expression of markers associated with residency in tissue (CD69 and CD103) (22) or with cellular activation (HLA-DR) (Figure 2A). In terms of HLA-DR expression, pathological biopsies showed significantly higher percentages of this molecule in total NK cells and in the CD56^+^CD16^–^ NK cell fraction compared with normal biopsies (*P* = 0.030 and *P* = 0.024; Figure 2, C and D, respectively). Further, both pathological and normal samples showed very high expression of CD69 in NK cells, primarily within the CD56^+^ NK subset (regardless of CD16 expression); however, this expression was higher in normal samples compared with pathological samples (*P* = 0.034 and *P* = 0.041; Figure 2, E and F, respectively). Last, when we analyzed CD103 expression together with CD69, we detected lower proportions of these markers in total NK cells from HSIL samples in comparison with normal or LSIL samples (*P* = 0.042 and *P* = 0.019, Figure 2G).

We additionally determined the effect of age, nadir CD4, time on cART, or presence of HR-HPV on the differences observed in the NK subsets based on pathology (Supplemental Figure 4). Most differences observed between normal and pathological biopsies were maintained, in particular the increase in HLA-DR expression and the decrease in CD69 associated with dysplasia, though statistical significance was limited in most comparisons of the post hoc analyses (Supplemental Figure 4). These results indicate that CD16^+^CD56^–^ NK cells and overall HLA-DR expression are augmented during pathology, while the expression of residency markers CD69 and CD103 is compromised in dysplastic environments. Overall, CD56 NK subsets appeared to be most affected by these changes, which seemed not to depend on age, nadir CD4, time on cART, or presence of HR-HPV16/18 genotypes.

### Potentially suppressive myeloid cell subsets are augmented in anal dysplasia.

**Figure 3:**
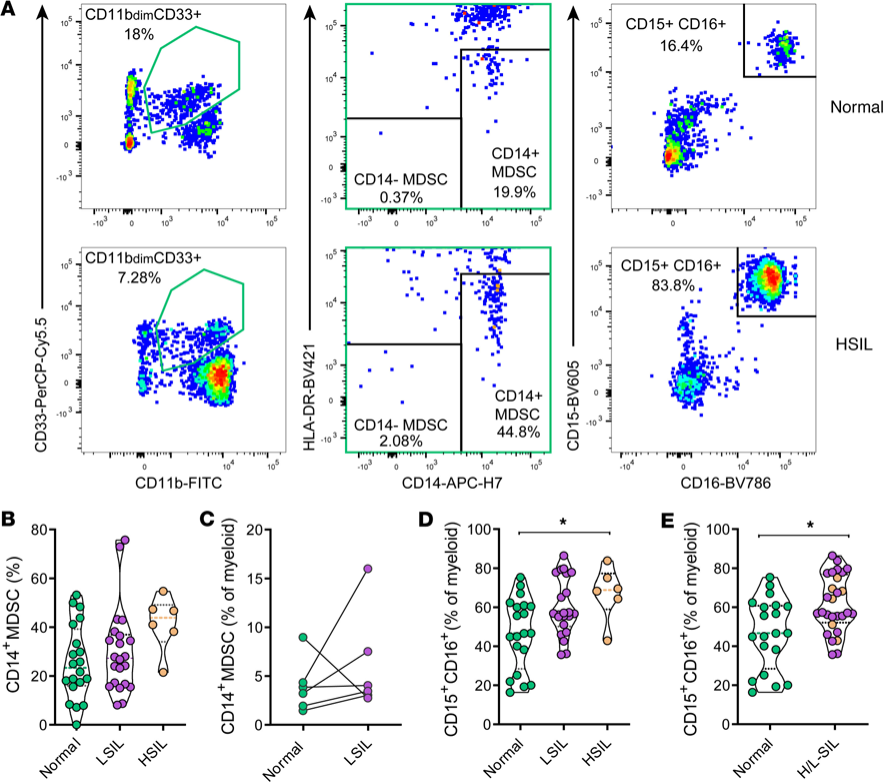
Frequency of myeloid populations associates with SIL. (**A**) Flow cytometry gating strategy used to quantify myeloid-derived suppressor cells (MDSCs) (out of CD11b^dim^CD33^+^, in green) and neutrophils (CD15^+^) expressing CD16 is shown for a nonpathological sample (top) and for a high-grade squamous intraepithelial lesion sample (HSIL, bottom). Sequential gating from left to right was used to determine 2 different MDSC subsets (CD14^–^HLA-DR^–^ and CD14^+^HLA-DR^dim/–^). (**B**) Frequency of CD14^+^ MDSCs out of the CD11b^dim^CD33^+^ myeloid gate in normal (green), LSIL (purple), and HSIL (brown) samples. (**C**) Frequency of CD14^+^ MDSCs out of total myeloid cells in paired normal and LSIL samples from the same individual analyzed by the Wilcoxon signed rank test. (**D** and **E**) Frequency of neutrophils (CD15^+^CD16^+^) out of the total myeloid fraction in (**D**) normal, LSIL, and HSIL or in (**E**) normal (in green) versus pathological (H/L-SIL, in purple; HSIL are highlighted in brown) samples. Data are represented as a violin plot; horizontal lines are median and interquartile range. Statistical comparisons using nonparametric Kruskal-Wallis test with Dunn’s post hoc test for multiple comparisons and Mann-Whitney *U* test for 2-group analyses are shown: *P < 0.05.

We additionally determined the frequency of several myeloid subsets, including a subset of potentially immune-tolerant cells, called myeloid-derived suppressor cells (MDSCs), and mature neutrophils (CD15^+^CD16^+^, Figure 3A) (23, 24). Of note, MDSCs were defined as CD11b^dim^CD33^+^ myeloid cells with either an HLA-DR^–^CD14^–^ or an HLA-DR^lo^CD14^+^ phenotype (24), as shown (Figure 3A). In certain samples, the percentage of MDSCs expressing CD14 was significantly increased with pathology: the HSIL group showed a median of 43.85 (IQR: 34.03–54.70), the LSIL group showed a median of 27.30 (IQR: 16.45–36.93), and the normal group had a median of 23.35 (IQR: 16.05–39.23). However, due to high variability within the normal samples, statistical significance was not achieved, with only a trend observed when compared to the HSIL group (*P* = 0.082, Figure 3B). Still, out of the 6 individuals with paired normal and LSIL samples, 5 of them exhibited an increase in CD14^+^ MDSCs in the LSIL sample compared with the nonpathological sample (Figure 3C). Regarding the evaluation of CD15^+^CD16^+^ neutrophils, we detected a gradual increase in their percentage associated with the severity of the SIL. There was a statistically significant difference between normal and HSIL samples (*P* = 0.047, Figure 3D), and this association became stronger when all pathological samples were grouped together and compared with normal samples (*P* = 0.012, Figure 3E). Post hoc analyses considering age, nadir CD4, time on cART, or presence of HR-HPV on the differences for myeloid subsets based on pathology were also performed (Supplemental Figure 5). Most differences observed between normal and pathological biopsies regarding CD14^+^ MDSCs were lost, but did not seem affected by these factors either, while CD15^+^CD16^+^ neutrophils were strongly increased in pathological samples from patients 45 years old or less, with nadir CD4 levels equal or inferior to 350 cells, with less than 10 years of cART, and without HPV16/18 genotypes (Supplemental Figure 5). Together, increased proportions of CD14^+^ MDSCs and mostly CD15^+^CD16^+^ neutrophils were found to be associated with dysplasia.

**Figure 4:**
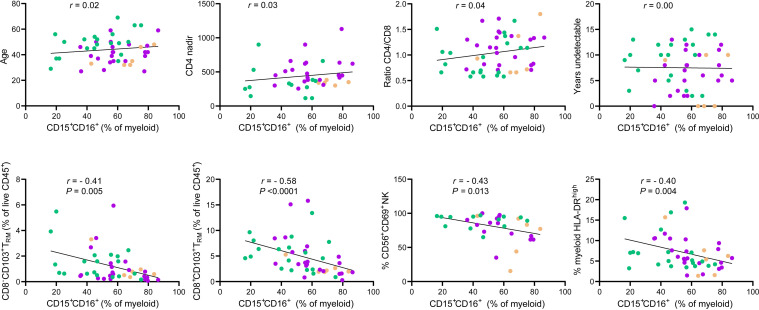
Correlation between clinical and immunological parameters and the frequency of anal CD15^+^CD16^+^ myeloid cells in anal samples from PWH. Correlation between the frequency of CD15^+^CD16^+^ out of the total myeloid fraction in normal (green), LSIL (purple), and HSIL (brown) anal samples and clinical parameters associated with the individual (top row from left to right: age, CD4 nadir, CD4/CD8 ratio, and years of having undetectable < 50 copies/mL) or with other immunological subsets determined by flow cytometry and identified as defined in Supplemental Figure 1 (bottom row from left to right: CD8^–^ T_RM_, CD8^+^ T_RM_, CD69^+^CD3^–^CD56^–^ NK, and HLA-DR^hi^ myeloid cells). Statistics were performed using nonparametric Spearman rank correlation and nonlinear regression.

Considering that the frequency of this myeloid subset expressing CD15*^+^*CD16*^+^* appeared as the best flow cytometry–derived subset to classify pathology, we additionally assessed the level of correlation between this parameter and other immune subsets and clinical parameters. The frequency of this subset out of the total myeloid fraction did not show any correlation with age, CD4 nadir, CD4/CD8 ratio, or the number of years under viral suppression for each individual (Figure 4). Nevertheless, there was a moderate negative correlation between this subset and the total frequency of CD8^–^ T_RM_ (*r* = –0.41, *P* = 0.005) as well as CD8*^+^* T_RM_ (*r* = –0.58, *P* < 0.001) in the same samples, the frequency of CD3^–^ CD56^–^ expressing CD69 (regardless of CD16 expression; *r* = –0.43, *P* = 0.013), and the frequency of myeloid cells expressing high levels of HLA-DR (*r* = –0.40, *P* = 0.004). In contrast, a positive correlation was observed between the total frequency of CD3^–^CD16*^+^* NK cells (regardless of CD56 expression) and CD15*^+^*CD16*^+^* myeloid cells (*r* = 0.33, *P* = 0.036, Supplemental Figure 6).

### CD15 epithelial staining as a complementary biomarker for diagnosis.

**Figure 5:**
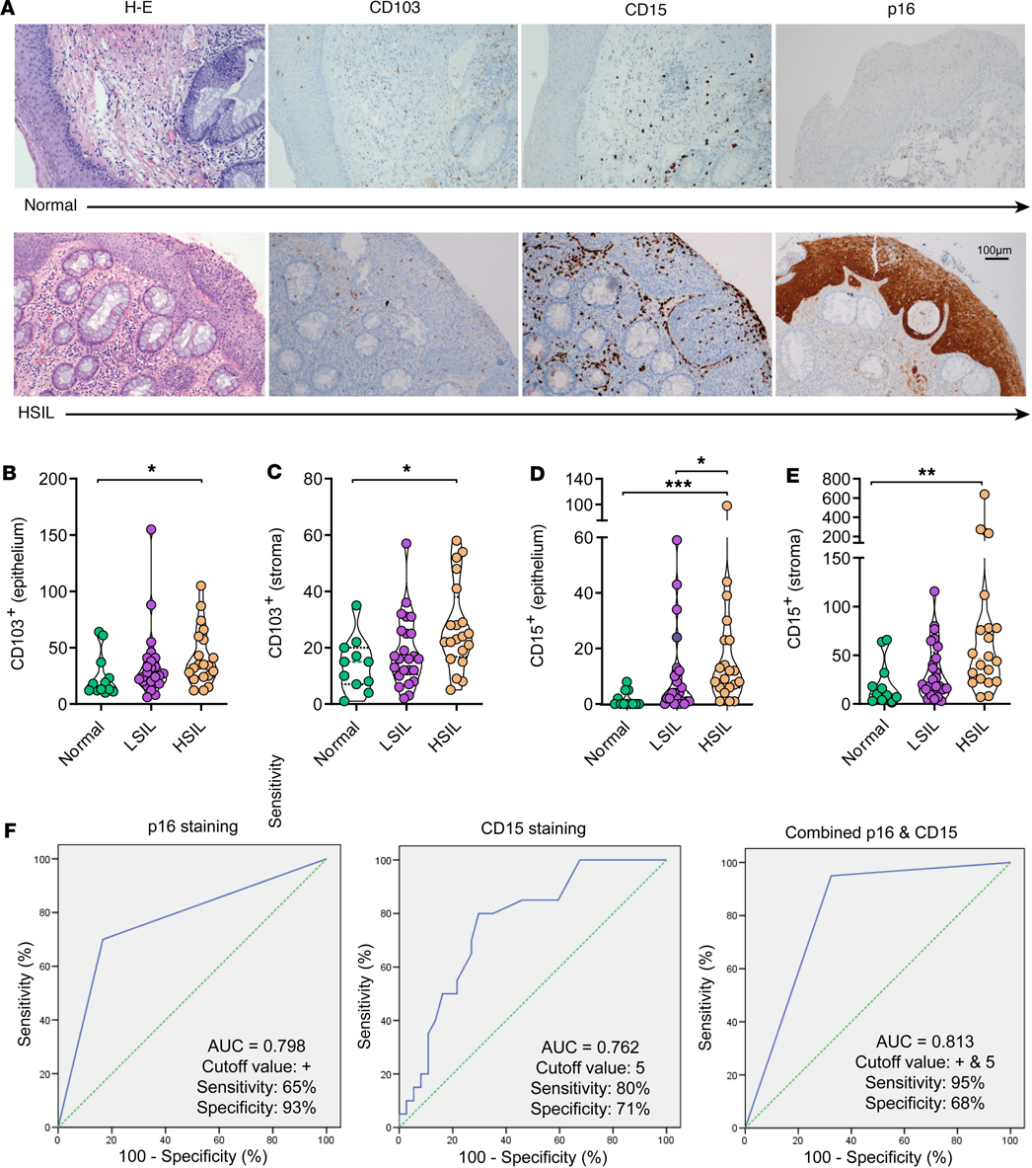
Detection of immunological biomarkers by immunohistochemistry. (**A**) Examples of microphotographs showing hematoxylin and eosin, CD103, CD15, and p16 staining of the intraepithelial compartment and the underlying lamina propria (stroma) of a normal (top) and a high-grade squamous intraepithelial lesion (HSIL, bottom) mucosal samples. Original magnification, ×20 (scale bar is indicated). (**B**–**E**) Quantification of the average number of positive cells detected per a median of 3 fields (range 1 to 7) at ×40 original magnification in the (**B**) epithelium or the (**C**) stroma for CD103 staining or in the (**D**) epithelium or the (**E**) stroma for CD15 staining in normal (green), LSIL (purple), and HSIL (brown) anal samples. Data are represented as a violin plot; horizontal lines are median and interquartile range. Statistical comparisons using nonparametric Kruskal-Wallis test with Dunn’s post hoc test for multiple comparisons are shown: *P < 0.05; **P < 0.01; ***P < 0.001. (**F**) Receiver operating characteristics curves showing the area under the curve (AUC), cutoff, sensitivity, and specificity values for CD15 and p16 staining and their combination. Green dotted line: theoretical performance of an efficacy biomarker equivalent to a coin toss. Blue line: actual performance of the results.

Based on our results, we selected CD103 and CD15 molecules for further validation of our findings through immunohistochemistry (Figure 5A). Our main objective was to determine their diagnostic value as individual pathology markers in comparison with p16, which is currently recommended to support the diagnosis of HSIL in the appropriate morphology context (16, 25). To this end, we obtained a new set of 57 archived tissue sections as the validation cohort, which did not show differences in clinical parameters between groups, except for HR-HPV genotypes (Table 2). CD103- and CD15-positive cells were individually counted within the epithelium or the underlying stroma. Unexpectedly, the average CD103 count within the epithelium and stroma of HSIL biopsies was higher compared with normal samples (Figure 5, B and C). These findings suggested that other subsets beyond T cells, such as NK cells and CD15^+^ neutrophils, as previously reported (22, 26), could potentially exhibit a higher frequency of CD103 expression in association with pathology. Indeed, the subsequent analysis of CD103 expression within the neutrophil CD15^+^CD16^+^ subset obtained from the flow cytometry data demonstrated an overall increase of this molecule in the pathological samples compared with the normal samples (*P* = 0.046, Supplemental Figure 7), suggesting their epithelial location. Actually, in line with these results, immunohistochemistry analyses evidenced an increase in CD15 counts in the epithelium and stroma of HSIL samples compared with both normal and LSIL samples (*P* = 0.0001 and *P* = 0.039, respectively, for the epithelium, and *P* = 0.0042 and *P* = 0.074, respectively, for the stroma; Figure 5, D and E).

Since the validation cohort showed significant differences in the presence of HR-HPV associated with pathology (Table 2), we also performed a post hoc analysis separating by the presence or not of HPV16/18 genotypes, as well as the clinical parameters analyzed beforehand for the discovery cohort. These analyses evidenced that HSIL samples consistently had increased numbers of CD15 in the epithelium and, less so, in the stroma, compared with LSIL and normal samples (Supplemental Figure 8). Regarding the presence of HPV16/18 genotypes, differences between HSIL and LSIL or normal biopsies were more obvious when these genotypes were absent (*P* = 0.019 and *P* = 0.002, respectively, for epithelium; and *P* = 0.029 and *P* = 0.022, respectively for stroma; Supplemental Figure 8D). Considering that when these genotypes were present only 2 samples remained normal, statistical significance was lost, but trends of lower CD15 detection in normal samples compared with pathological samples remained (Supplemental Figure 8D).

Further, in our study, p16 staining correlated with HSIL diagnosis with a sensitivity of 65% and a specificity of 93% (AUC 0.798, Figure 5F). In comparison, a threshold of more than 5 positive CD15 cells in the epithelium had a sensitivity of 80% and a specificity of 71% (AUC 0.762, Figure 5F). Importantly, the combination of both biomarkers, meaning a threshold of more than 5 positive CD15 cells and a positive p16 staining, showed a sensitivity of 95% and a specificity of 68% (AUC 0.813, Figure 5F).

Considering that the majority of lesions diagnosed as HSIL in the validation cohort underwent subsequent treatment, we aimed to determine the predictive value of CD15 staining regarding the response to treatment. Out of all the pathological samples that had a follow-up biopsy performed at the same previous site (19 out of the 20), 3 samples remained classified as HSIL, 10 samples showed a decrease in severity to LSIL, and 6 samples completely responded to treatment and were classified as normal biopsies. When comparing the quantification of CD15-positive cells in the epithelium between the samples that completely responded to treatment (regressed to normality) and those that remained as an HSIL diagnosis or decreased to an LSIL, a trend toward lower numbers of this biomarker in the pathological samples that regressed was observed (Supplemental Figure 9). Indeed, it is noteworthy that samples negative for p16 (highlighted as triangles in Supplemental Figure 9) were observed in all groups with different treatment outcomes. This observation suggests that quantifying CD15 in the epithelium could potentially serve as a more reliable indicator for predicting the response to treatment, pending further validation.

**Figure 6:**
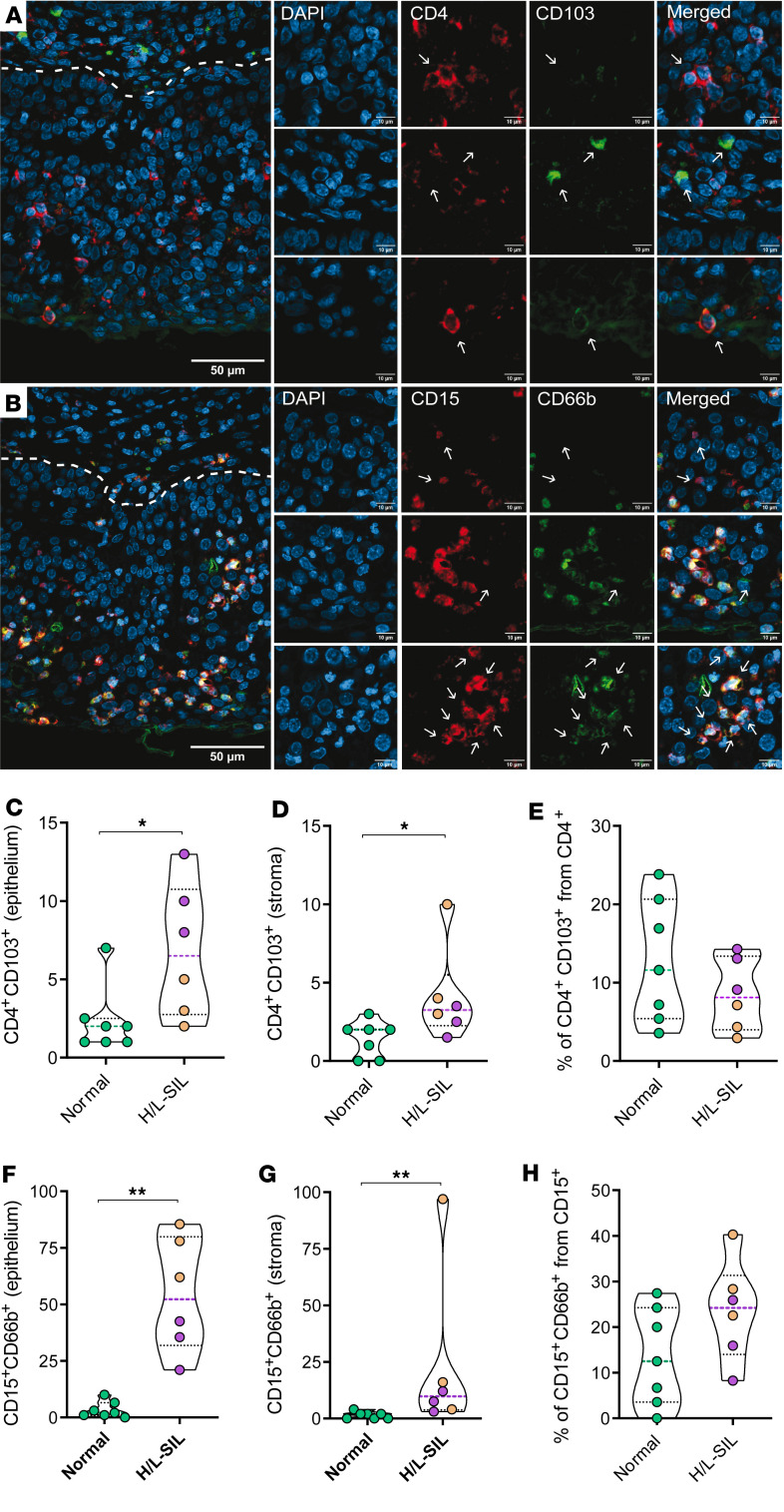
Detection of CD4/CD103 and CD15/CD66b by IF. (**A**) Microphotograph showing anti-CD4 (red) and anti-CD103 (green) in the epithelium and the underlying stroma, indicated by a white dashed line, of a high-grade squamous intraepithelial lesion (HSIL) sample. Right panels depict magnified views of samples captured in various channel acquisition colors using a ZEISS LSM 980 confocal microscope with ×40 oil immersion Plan-Apochromat objectives (NA 1.3): top row denotes single CD4^+^ cells, middle row single CD103^+^ cells, and bottom row double CD4^+^CD103^+^ cells (all indicated by arrows). (**B**) Microphotograph showing anti-CD15 (red) and anti-CD66b (green) in the epithelium and the underlying stroma, indicated by a white dashed line, of an HSIL sample. Right panels depict magnified views, obtained as in **A**, of single CD15^+^ cells in top row, single CD66b^+^ cells in middle row, and double CD15^+^CD66b^+^ cells in bottom row (all indicated by arrows). (**C**–**G**) Median number of double CD4/CD103-positive (**C** and **D**) and CD15/CD66b-positive (**F** and **G**) cells detected per a median of 6 fields (range 2 to 15) at ×25 original magnification in the (**C** and **F**) epithelium or the (**D** and **G**) stroma in normal (green) and pathological (H/L-SIL, in purple; HSIL are highlighted in brown) samples. (**E** and **H**) Percentage of double positive out of total CD4 (**E**) and CD15 (**H**). Data are represented as a violin plot; horizontal lines are median and interquartile range. Statistical comparisons using nonparametric Mann-Whitney *U* test for 2-group analyses are shown: *P < 0.05; **P < 0.01.

Last, to verify the findings from the immunohistochemistry analyses and relate them to the original flow cytometry data obtained, we performed immunofluorescence (IF) analyses in an additional small subset of biopsies from the initial discovery cohort. Thus, we performed costaining of CD4 and CD103 cells (Figure 6A) and of CD15 and CD66b (a common marker to identify neutrophils, ref. 27) cells (Figure 6B) and quantified single and double-positive cells in the epithelium and the lamina propria. These analyses showed that the median counts of CD4^+^ and CD103^+^ positive cells in both epithelial and stromal areas were higher for pathological compared with nonpathological samples (*P* = 0.022 and *P* = 0.025, respectively; Figure 6, C and D). However, when calculating the proportion of double CD4^+^CD103^+^ from the total CD4 counts, pathological samples showed, in general, low percentages (Figure 6E). In addition, quantification of double CD15- and CD66b-positive cells verified higher levels of double-positive cells located in the epithelium and stroma of pathological compared with normal biopsies (*P* = 0.001 and *P* = 0.002, respectively; Figure 6, F and G), but in this case, the proportion of CD15^+^CD66b^+^ cells with respect to CD15^+^ cells remained in the high range in association with dysplasia (Figure 6H). These results indicate that there is an overall increase or infiltration of immune cells in pathological areas, already suggested by the high median count of viable hematopoietic CD45^+^ cells retrieved from pathological biopsies (Tables 1 and 2). Indeed, single CD4^+^ T cell quantification in tissue slides from pathological samples by IF, which was markedly increased in pathological samples (*P* = 0.001, for both epithelium and stroma; Supplemental Figure 10A), was also accompanied by a higher CD8^–^CD3^+^ event count median in the biopsies from the flow cytometry data, with a median of 975 (IQR: 381–2,036) for the HSIL, of 381 (IQR: 173–860) for the LSIL, and of 240 (IQR: 137–815) for normal samples. Consequently, there was a significant negative correlation within individual samples between the frequency of CD103^+^CD8^–^ T_RM_ out of the total live CD45^+^ fraction measured by flow cytometry and the quantification of CD4^+^CD103^+^ cells (*r* = –0.78, *P* = 0.003; Supplemental Figure 10E). The explanation to this apparent contradiction is the difference between these techniques in terms of quantification as well as other phenotypic markers included to identify subsets. Thus, while an overall CD4^+^ count increase is observed and quantified by techniques that provide absolute numbers, such as IF, the proportion of cells that express CD103 out of this subset is low in pathological samples, shown by IF and also flow cytometry, in which CD69 was concomitantly assessed to identify the proportion of T_RM_ out of CD45^+^ live cells. In contrast, there was a positive correlative trend between the frequency of CD15^+^CD16^+^ cells out of the myeloid fraction and the quantification of CD15^+^ CD66b^+^ cells (*r* = 0.55, *P* = 0.055; Supplemental Figure 10F), which was significant when considering CD15^+^ positive cells only (*r* = 0.68, *P* = 0.012; Supplemental Figure 10G). In summary, the findings from immunohistochemistry and IF verified the presence of CD15^+^ neutrophils associated with dysplasia in the anal mucosa. Moreover, the identification of these cells in the epithelium serves as a valuable pathological marker in this context.

## Discussion

Persistent infections share immunological features with the tumor environment, where the balance between effector mechanisms and suppressive or inflammatory populations is disrupted. In this sense, the anal SIL may be of particular interest, since it may combine a persistent viral infection with a tumor microenvironment. However, detailed assessment of relevant resident or infiltrated immune subsets within affected dysplastic areas has largely been missing for transitional anal tissue. Overall, we identify a potentially enriched immunosuppressive environment associated with pathological samples. Importantly, our findings highlight CD15 as an immunological marker that could contribute to an improved diagnosis of HSIL.

Effective resident immunity, including T_RM_ subsets, play essential roles in controlling persistent infections (28, 29). E6-specific CD4^+^ T cell responses may be associated with recent HSIL regression (14). In contrast, skewing of HPV-specific T cells from an effector Th1 to a Th2 profile or increased expression of programmed cell death 1 in infiltrating CD8^+^ T cells in patients with venereal warts may suggest suppressed effector immunity (30). Further, CD69^+^CD103^+^ T_RM_-like cells accumulate in various human solid cancers, where they have been associated with improved disease outcome and patient survival (31). In anal dysplastic lesions, overall CD8^+^ T cell infiltration or expansion has been reported (19, 30), which concurs with our observation of a higher frequency of CD8^+^ T cell lymphocytes in HSIL samples compared with concurrent normal mucosa from the same individual. However, expression of CD103 within this compartment was lower in dysplastic compared with nonpathological samples. In this sense, a persistent depletion of CD4^+^ T_RM_ phenotypes from the mucosal compartments has been reported in PWH who have been treated during chronic infection (11, 12). Considering that CD4^+^ T_RM_ promote the development of CD103-expressing CD8^+^ T_RM_ in certain tissues (32), their generation may also be compromised in these patients. Still, in our cohort, other aspects associated with the dysplastic environment may have a greater impact, since all PWH included were cART treated during the chronic phase. In fact, factors like transforming growth factor-β (TGF-β) availability, which is essential for CD103 expression and T_RM_ development; epithelial dysfunction; and chronic antigen exposure may affect CD103 expression (33–35). While we could speculate that the TGF-β signaling is affected within pathological areas (36), our data showed the opposite for mature neutrophils, which showed higher levels of CD103 expression in those areas, with more retention within the epithelium. Thus, other mechanisms, such as an impaired CD38 signaling, autocrine secretion, or the availability of TGF-β1 for T cells, could be at play (37, 38).

NK cells are also known for their key role in viral and tumor clearance, including resident memory NK cells (39). High expression of canonical markers, such as CD69 and CD103, in CD56^+^ NK cells identify resident memory NK cells in specific tissues, such as the liver, lung, or uterus (22, 39, 40). In anal tissue, the expression of CD69 within the CD56 fraction of nondysplastic samples was generally over 90%. However, HSIL and LSIL biopsies presented lower proportions of CD69^+^CD56^+^ NK cells, and HSIL samples of total CD69^+^CD103^+^ NK cells, suggesting again that the shrinkage of the resident lymphocyte effector compartment may contribute to the lack of control of the nascent dysplasia. In contrast, HLA-DR expression and a high proportion of CD16^+^ NK cells were associated with pathology. HLA-DR indicates activation in several lymphocyte subsets, and an accumulation of HLA-DR–expressing NK cells at sites of inflammation has been reported (41). Regarding CD16^+^ NK cells, this subset includes CD56^–^CD16^+^ NK cells, which have been shown to expand during viral infections to form an anergic population with impaired cytotoxic activities (39). Our results are somewhat consistent with NK cell deficiency affecting CD56 populations, which renders patients more susceptible to HPV and herpes simplex virus infection and HPV-related diseases (42). In agreement, a general decrease of CD56^+^ NK cells has been associated with cervical dysplasia in HPV/HIV-coinfected women (43), while presence of CD56^+^ cells has been associated with increased overall survival in squamous cell carcinoma of the oropharynx, independent from HPV (44).

Engaging effector mechanisms may be of particular importance in individuals infected with various persistent viruses, such as HPV and HIV, which exploit immune modulation mechanisms from the host to induce immune tolerance and limit viral clearance (45, 46). Indeed, the local inflammatory state generated by chronic infection, including molecules like granulocyte colony-stimulating factor (47), could induce accumulation of undesired suppressive cells, such as MDSCs, as reported (48, 49). Although previous studies suggest that myeloid cells might be displaying an immunosuppressive effect in HPV-induced malignancies (47, 50, 51), the mechanisms responsible for the various immune-related defects observed in these patients remain unclear. Furthermore, the so-called mature CD15 neutrophils, identified by high expression of CD16 and expression of CD66b (27), may also play a controversial role. They have been linked to inflammatory conditions (52), with increased numbers in patients with periodontitis (53) or vaginitis (54). However, they might be suppressing T cells even in the context of inflammation (52), impairing and exerting strong T cell immunosuppression (23, 47, 55), even in tumor microenvironments (56). The fact that CD15^+^ granulocytic MDSCs and neutrophils share expression of CD15^+^, CD16^+^, and CD66b^+^ molecules indicates that only functional assays would confirm their immunosuppressive properties (57, 58), and future research in this area is warranted. Still, both CD14^+^ MDSCs and CD15^+^CD16^+^ mature neutrophils are known to be key hallmarks of tumor inflammation and immune suppression, subsets that are also involved in chronic infections (23, 24). Thus, the fact that we observed a gradual increase of these subsets from normal to HSIL samples suggests that an immunosuppressive environment may favor dysplasia progression. Actually, a link between systemic amplification of myeloid cells and the detrimental effects of these cells on CD8^+^ T cell activation and recruitment into the tumor microenvironment has been proposed (48).

Importantly, immunohistochemistry and IF analyses verified the infiltration of CD15^+^ neutrophils in the anal mucosa associated with dysplasia, showing its potential value as a biomarker for pathology staging. Substantial disagreement exists among experienced pathologists in diagnosing SIL by H&E morphology, which is the gold standard (16). In this sense, addition of p16 immunohistochemistry increases interobserver agreement, yet discrepancy remains considerable regarding intermediate lesions (16). Thus, it is crucial to identify additional markers that can help minimize the misdiagnosis of HSIL and avoid unnecessary treatments. Moreover, the identification of reliable markers is essential for accurately identifying individuals with precancerous lesions who are at risk of disease progression. In our study, the determination of CD15 and p16, which shared similar technical complexity, since they were both detected by immunohistochemistry, exhibited a similar capacity to reliably detect dysplasia. Thus, in cases in which p16 was negative, the determination of epithelial CD15 staining, based on the established threshold, could help differentiate between HSIL and LSIL. Further, the fact that differences were stronger in samples negative for HR-HPV genotypes provides additional value to follow up these patients with elevated numbers of CD15 in their epithelium. It should also be noted that we observed an inverse association between the epithelial infiltration of CD15 in the HSIL samples and the response to treatment, which was not observed with p16. Of note, while we did not include patients with anal cancer, other works have highlighted the importance of neutrophils and CD15 expression in cancer as biomarkers of progression and response to treatment (59, 60). Thus, future larger studies should aim to validate the utility of CD15 staining as a complementary measurement for the diagnosis of L/HSIL, or even as a prognostic marker, in particular if this marker can be eventually assessed by noninvasive techniques.

It is important to note that this study is limited by the number of samples, in particular within the HSIL group in the discovery cohort, which was restricted by the complexity of the analyses and the impossibility of preselecting samples based on the degree of dysplasia. However, CD15 results were verified in the validation cohort, which included more homogeneous groups of samples. Of note, as another limitation, 8 patients had different time point samples in both cohorts. Besides, because dysplasia development takes years to progress, we lack the longitudinal analyses that would inform on the actual predictive value of these markers regarding lesion evolution to cancer. Future studies will address the function and interactions between these resident immune cells to define key populations in anal cancer precursor progression. In summary, our results expand current knowledge of mucosal immunity in anal dysplasia. The identification of CD15 as a potential complementary biomarker for HSIL diagnosis suggests its potential application in improving diagnostic tools and may have implications for the development of targeted immunotherapeutic strategies for this condition.

## Methods

### Sex as a biological variable.

This study involved MSM with HIV. Only MSM with HIV were included because they are the group with the highest risk of anal cancer and in whom screening for anal dysplasia is recommended. Since our study focused on characterizing the immunological environment where lesions develop to identify biomarkers that may contribute to the diagnosis of anal dysplasia, it was advisable to start with the highest risk group. Further studies would be necessary to determine if the findings are applicable to women or other groups of persons at risk.

### Study design and patient cohorts.

The Anal Dysplasia Unit at the University Hospital Vall d’Hebron (HUVH, Barcelona, Spain) was created in May 2009 and attends more than 1,000 MSM with HIV. Anal screening includes anal liquid cytology and HPV determination, a high-resolution anoscopy (HRA) and, when necessary, anal biopsies, as previously described (61). Patients undergoing anal biopsies as part of the screening program were offered to participate in the study with the following inclusion criteria: patients on cART, with HIV viral suppression, and without any anal sexually transmitted disease or treatment for HSIL in the last 6 months. Patients were included prospectively for the initial immunological and histological analyses, while for the validation of the results by immunohistochemistry, patients were recruited retrospectively from available histological samples.

### Sample collection.

Cytology was obtained by introducing a Dacron swab 3–5 cm into the anal canal and softly rotating it. The swab was introduced into 20 mL of PreservCyt/ThinPrep Pap test solution (Cytyc Iberia S.L.) and shaken for 30 seconds. This sample was used to carry out the cytological analysis and HPV testing (61). Single or multiple anal biopsies were taken from individual patients in the same screening session if HRA revealed an abnormal area or in areas that were previously treated to determine treatment efficacy. For a single biopsy, an immunological and histological study was carried out simultaneously. An expert pathologist classified samples using the terminology and morphological criteria published in the Lower Anogenital Squamous Terminology project: benign, LSIL, and HSIL (16, 62).

### HPV detection.

DNA was extracted from cytology-derived cell suspensions using the QIAamp Viral DNA minikit (QIAGEN). Specific sequences of papillomavirus were amplified by specific protocol CLART Genomic HPV-2 in accordance with the manufacturer’s instructions. Detection of HPV genotypes 16, 18, 31, 33, 35, 39, 45, 51, 52, 56, 58, 59, 68, 73, and 82 were considered as high risk.

### Immunological cell phenotyping by cytometry.

Fresh anal tissue samples of ≈8 mm^3^ were collected in antibiotic-containing RPMI 1640 medium. Samples were enzymatically digested with 5 mg/mL collagenase IV (Gibco, Thermo Fisher Scientific), and the resulting mononuclear cell suspension was washed twice and stained for viability with Live/Dead Aqua (Invitrogen, Thermo Fisher Scientific) at room temperature for 30 minutes in PBS. Cells were then washed with PBS and surface-stained using a 13-color flow cytometry panel (Supplemental Table 1). After fixation, all events were acquired using a BD LSRFortessa flow cytometer, and data were analyzed with FlowJo vX.0.7 software (TreeStar). We established a minimum of 1% of CD45^+^ cells from the total stored events as well as additional minimums per subset count to consider the sample for the analyses: 100 events for CD3^+^ T cell lymphocytes, 50 events for CD3^–^ lymphocytes, and 100 events for myeloid cells.

### Immunohistochemistry.

We analyzed CD103-positive (Abcam ab129202) and CD15-positive (Ventana 05266904001) cells by immunohistochemistry to assess their value as a pathology marker in archival specimens from anal biopsies obtained from the validation cohort. Formalin-fixed, paraffin-embedded anal samples of 3 μm sections were deparaffinized, rehydrated, and stained using optimal dilutions of monoclonal antibodies (Supplemental Table 1). The staining was performed following the protocol of the ultraView Universal DAB kit for Ventana Benchmark ultra. Mononuclear cells with a dark brown cytoplasmic signal were recorded as positive cells. Since intensity of the staining was homogeneous, the H score (an indicator of the intensity and proportion of the biomarker identified) was not used. Positive cells within the squamous epithelium and underlying stroma of the whole sample were manually counted using light microscopy (Olympus BX43) at ×40 original magnification by 2 independent pathologists. Sections were examined, avoiding lymphoid follicles of the stromal areas when present. The average number of positive cells from a median of 3 fields was reported for all the markers except for p16 (Ventana 05266904001), which was considered positive or negative based on the staining at the nuclear level of the squamous epithelial cells.

### IF.

Formalin-fixed, paraffin-embedded tissue slides underwent overnight deparaffinization at 65°C, followed by xylene and ethanol dilutions and fixation in 10% neutral buffered formalin. For CD103 (Abcam ab129202) and CD4 (Ventana 05552737001) staining, slides were placed in pH 9 antigen retrieval at 95°C for 30 minutes. After cooling, slides were blocked with 1× Block Opal buffer for 10 minutes, followed by incubation of CD103 (1:200) for 1 hour after TBS/Tween washing. Subsequently, 1× Opal Anti-Mouse + Rabbit HRP was applied and incubated for 45 minutes at room temperature. This allowed the Opal signal to be generated, using a 1:100 dilution of Opal Fluorophore 520 reagent in 1× Plus Manual Amplification Diluent, according to the manufacturer’s instructions (AKOYA Biosciences, NEL811001KT). CD103 fluorophore stripping was performed for 30 minutes at 95°C and pH 9, simultaneously working as the antigen retrieval step for CD4. Washes and incubations were performed as described before. However, the working dilution for CD4 was 1:25, and Opal Fluorophore 690 was used for signal generation. After CD4 stripping (performed as for CD103), the slides were counterstained with DAPI (1:1,000) for 7 minutes, washed, and mounted with Fluoromount-G (Invitrogen, Thermo Fisher Scientific). Staining with CD66b (Abcam ab197678) and CD15 (Abcam ab241552) was conducted as described above, with retrieval buffers at pH 6 and an antibody dilution of 1:25 for both markers. Images were initially captured in ×20 original magnification fields using a wide-field multidimensional Thunder microscope (Leica) for subsequent analyses. Quantification of epithelial and stromal single- and double-positive cells per sample was processed using ImageJ (NIH), in which binary masks for each marker were previously established. Confirmatory analyses and images at ×25 and ×40 original magnification were taken with a confocal microscope, ZEISS LSM 980, at a resolution of 2,048 × 2,048 pixels.

### Statistics.

Comparisons were performed between the 3 histological groups (normal, LSIL, and HSIL) as well as between 2 groups (normal versus pathological samples, which combined LSIL and HSIL samples) to increase statistical power. Statistical analysis was conducted using GraphPad Prism software. All tests assumed normal distribution and were 2 sided. Nonparametric Kruskal-Wallis test with Dunn’s post hoc test for multiple comparisons and Mann-Whitney *U* test or χ^2^ test were used for the unpaired analyses of 3 and 2 groups, respectively. For patients with paired normal and LSIL samples, we employed the Wilcoxon signed-rank test. Sensitivity, specificity, and the area under the receiver operating characteristic curve of potential biomarkers to detect HSIL were also determined in the validation cohort.

### Study approval.

Written informed consent for sample collection and use of information available in the medical records was obtained from all patients included. This study was performed in accordance with the Declaration of Helsinki and approved by the Institutional Review Board (PR(AG)240/2014) of the HUVH.

### Data availability.

All data associated with this study are present in the paper or the supplement, and raw data are included in the Supporting Data Values file.

## Author contributions

ABM, CM, NM, and AAG performed tissue processing and flow cytometry analyses. ABM, JC, and SL performed histology, immunohistochemistry, and IF analyses. JB, AC, JNGP, and VF collected samples and patient data. ABM, CM, MJB contributed to data analyses and discussion. JB and MG conceived and supervised the study and wrote the manuscript. All authors contributed to refinement of the study protocol and approved the final manuscript.

## Supplementary Material

Supplemental data

Supporting data values

## Figures and Tables

**Table 1:**
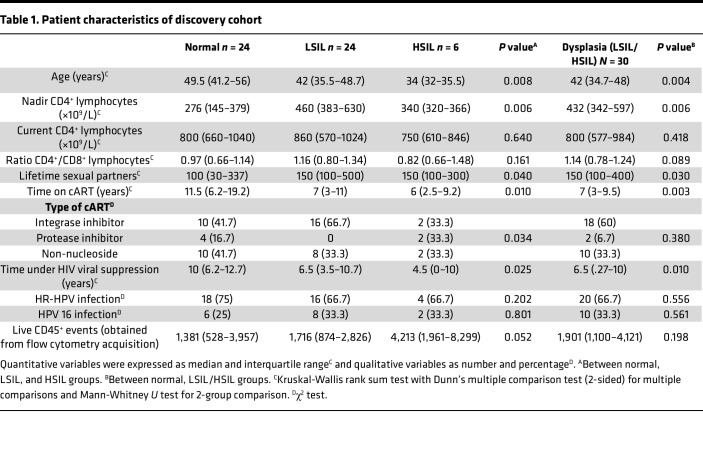
Patient characteristics of discovery cohort

**Table 2:**
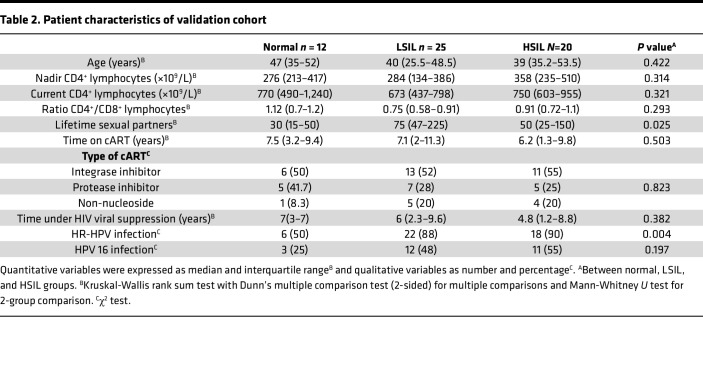
Patient characteristics of validation cohort

## Version 1 06/20/2024

In-Press Preview

## Version 2 08/08/2024

Electronic publication
