# Supplementary material for: Intraepithelial CD15 infiltration identifies high-grade anal dysplasia in people with HIV
Source: JCI Insight. 2024 Jun 20;9(15):e175251. doi: 10.1172/jci.insight.175251 (PMC11383605; doi:10.1172/jci.insight.175251)
Supplement: Supplemental data [file jciinsight-9-175251-s069.pdf]

Supplementary Data

Intraepithelial CD15 infiltration identifies high grade anal dysplasia in people with HIV

Burgos et al.

Supplementary Table 1. Anti-human antibodies used for flow cytometry, immunohistochemistry and immunofluorescence.

Antibody	Fluorochrome	Clone	Commercial source
**Flow Cytometry**			
CD3	PE-Cy7	SK7	BD Biosciences
CD33	PerCP-Cy5.5	WM53	BioLegend
CD11b	FITC	M1/70	BioLegend
CD45	Alexa700	HI30	BioLegend
CD56	PE	B159	BD Biosciences
CD14	APC-H7	MφP9	BD Biosciences
CD8	APC	RPA-T8	BD Biosciences
CD16	BV786	3G8	BD Biosciences
CD103	BV650	Ber-ACT8	BD Biosciences
CD15	BV605	W6D3	BD Biosciences
CD20	V500	L27	BD Biosciences
HLA-DR	BV421	G46-6	BD Biosciences
**Immunohistochemistry**			
CD15	NA	Mouse MMA	Ventana
CD103	NA	Rabbit EPR4166(2)	Abcam
CINtec® p16	NA	Mouse E6H4	Ventana
**Immunofluorescence**			
CD15	NA	Mouse 135B	Abcam
CD103	NA	Rabbit EPR4166(2)	Abcam
CD66b	NA	Rabbit pAb	Abcam
CD4	NA	Rabbit SP35	Ventana

NA not applicable

Supplementary Fig. 1

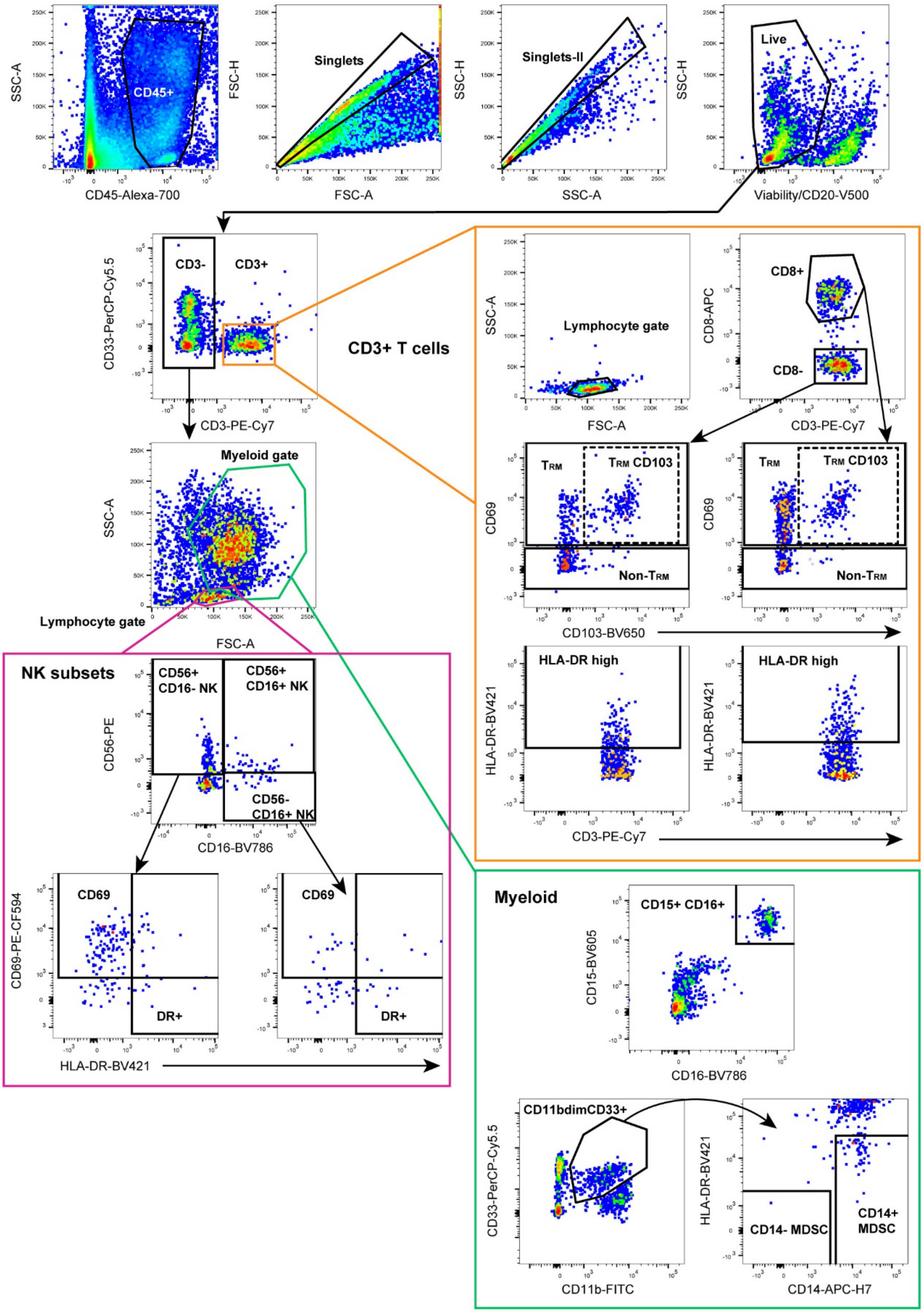

Figure S1. Flow cytometry gating strategy used to quantify the frequencies of immunological subsets of study in anal tissue samples. Sequential gating in the top plots (from left to right) allowed selecting single live hematopoietic non-B cells ($CD45^+$ singlets, $CD20^-$ viability dye^-). Secondly, the expression of CD3 was used to determine $CD3^+$ T cells ($CD3^+$, in orange) and natural killer (NK) or myeloid ($CD3^-$) subsets. Within the $CD3^+$T cell linage, $CD8^-$ and $CD8^+$ populations were gated and their sub-populations T_{RM}, Non-T_{RM} and T_{RM} $CD103^+$ were determined by the expression of CD69 and CD103, as well as, activated or non-activated by the expression of HLA-DR. Based on size and cellular complexity the NK (pink) and myeloid (green) linages were determined. NK lymphoid subpopulations (left bottom box, pink) were defined as $CD56^+CD16^-$, $CD56^+CD16^+$, or $CD56^-CD16^+$and their expression of CD69 (residency and/or activation) and HLA-DR (activation) determined. Within the myeloid linage (right bottom box, green), we gated mature neutrophils determined as $CD15^+CD16^+$, as well as putative myeloid derived suppressor cells (MDSC, defined by $CD11b^{dim}CD33^+$), which were sequentially gated as HLA-DR^-/$CD14^-$ or HLA-DR^{dim}/$CD14^+$ MDSCs.

Supplementary Fig. 2

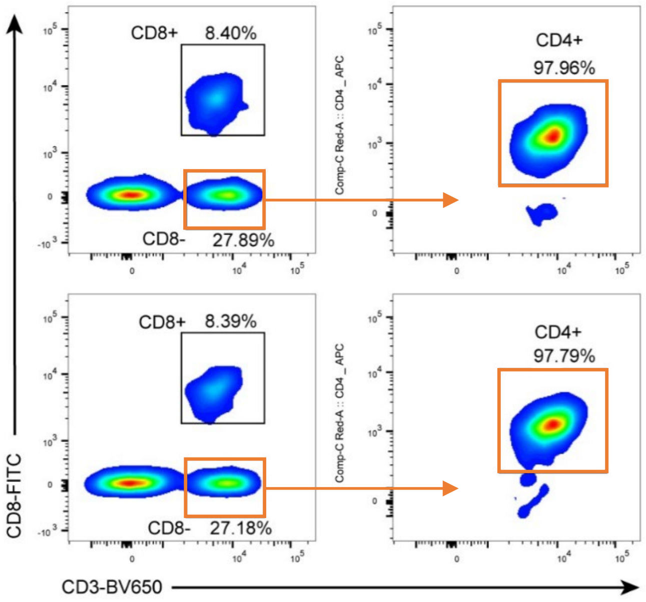

Figure S2. CD4$^+$ expression within CD8$^-$ CD3$^+$ T cells. The frequency of CD4$^+$ cells out of CD8$^-$ CD3$^+$ T lymphocytes, highlighted in orange, was evaluated in two additional anal samples.

Supplementary Fig. 3

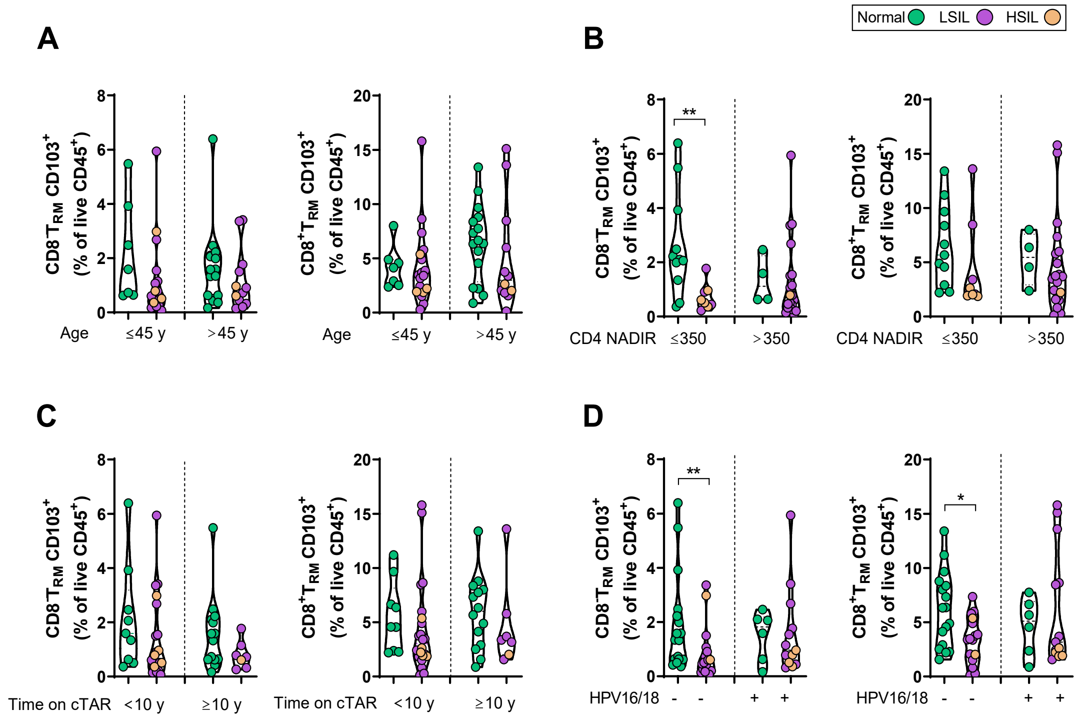

Figure S3. Differences in T_{RM} CD103$^+$ lymphocyte populations associated to squamous intraepithelial lesion by patient characteristics. Frequency of $CD8^-T_{RM}$ CD103$^+$ and $CD8^+T_{RM}$ CD103$^+$ lymphocytes in normal and pathological samples, including those with low-grade squamous intraepithelial lesions (LSIL) and high-grade squamous intraepithelial lesions (HSIL) by: **A**, age (≤45 or >45 years old); **B,** CD4$^+$ Nadir count (≤350 or >350 x10^9/L); **C,** Time on cART (<10 or ≥10 years of treatment); **D,** presence of HR-HPV genotypes (HPV-16 and HPV-18 negative or HPV-16 and HPV-18 positive). Data are represented as a violin plot representing normal patients (green) *versus* pathological (LSIL, in purple; HSIL highlighted in brown); horizontal lines are median and interquartile range. Statistical comparisons within groups using Mann-Whitney test are shown: *$p<0.05$; **$p<0.01$.

Supplementary Fig. 4

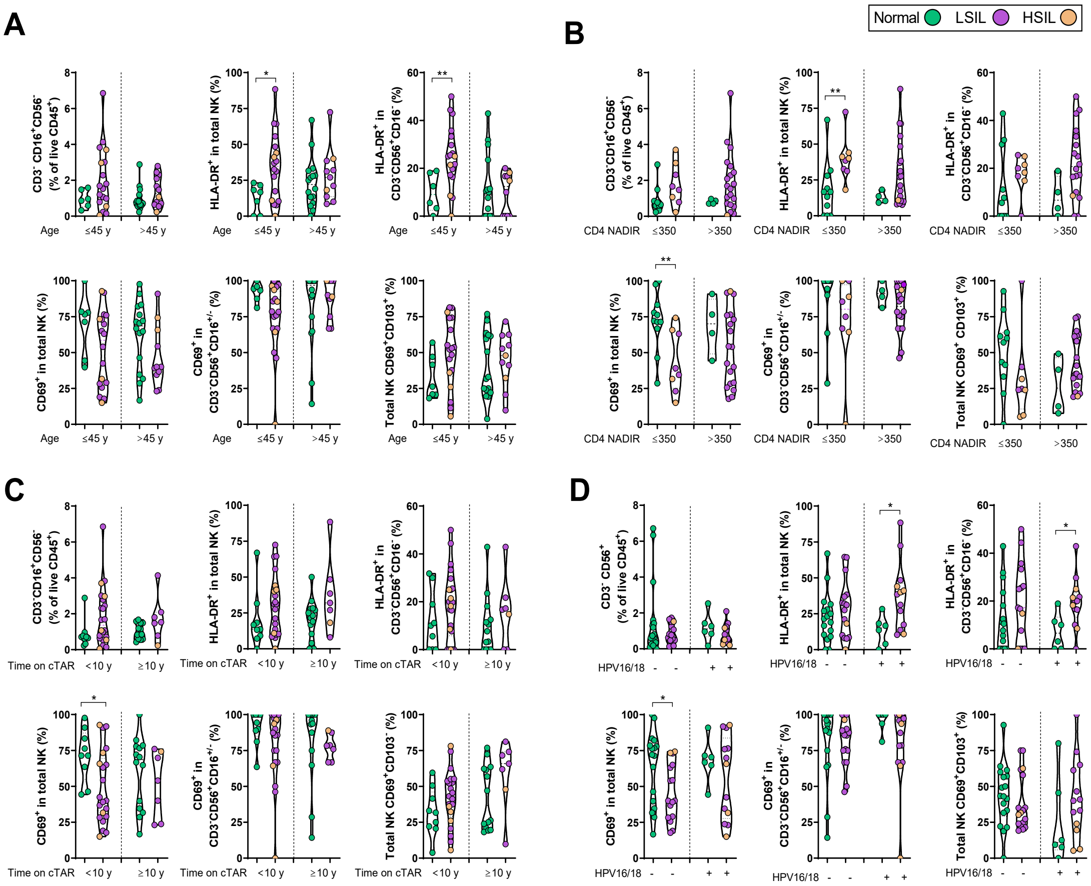

Figure S4. Differences in Natural Killer lymphocyte populations associated to squamous intraepithelial lesion by patient characteristics. Frequency of $CD3^{-}CD16^{+}CD56^{-}$ natural killer (NK) cells out of living $CD45^{+}$ cells; frequency of $HLA\text{-}DR^{+}$ out of total NK cells; frequency of $HLA\text{-}DR^{+}$ out of $CD3^{-}CD56^{+}CD16^{-}$; frequency of $CD69^{+}$ in total NK cells; frequency of $CD69^{+}$ in $CD3^{-}CD56^{+}CD16^{+/-}$; frequency of $CD69^{+}CD103^{+}$ NK cells out of total NK cells in normal and pathological samples, including those with low-grade squamous intraepithelial lesions (LSIL) and high-grade squamous intraepithelial lesions (HSIL) by: **A**, age (≤45 or >45 years old); **B,** Nadir $CD4^{+}$ count (≤350 or >350 x10^{9}/L); **C,** Time on cART (<10 or ≥10 years of treatment); **D,** presence of HR-HPV genotypes (HPV-16 and HPV-18 negative or HPV-16 and HPV-18 positive). Data are represented as a violin plot representing normal patients (green) *versus* pathological (LSIL, in purple; HSIL highlighted in brown); horizontal lines are median and interquartile range.-Statistical comparisons within groups using Mann-Whitney test are shown: *p<0.05; **p<0.01.

Supplementary Fig.5

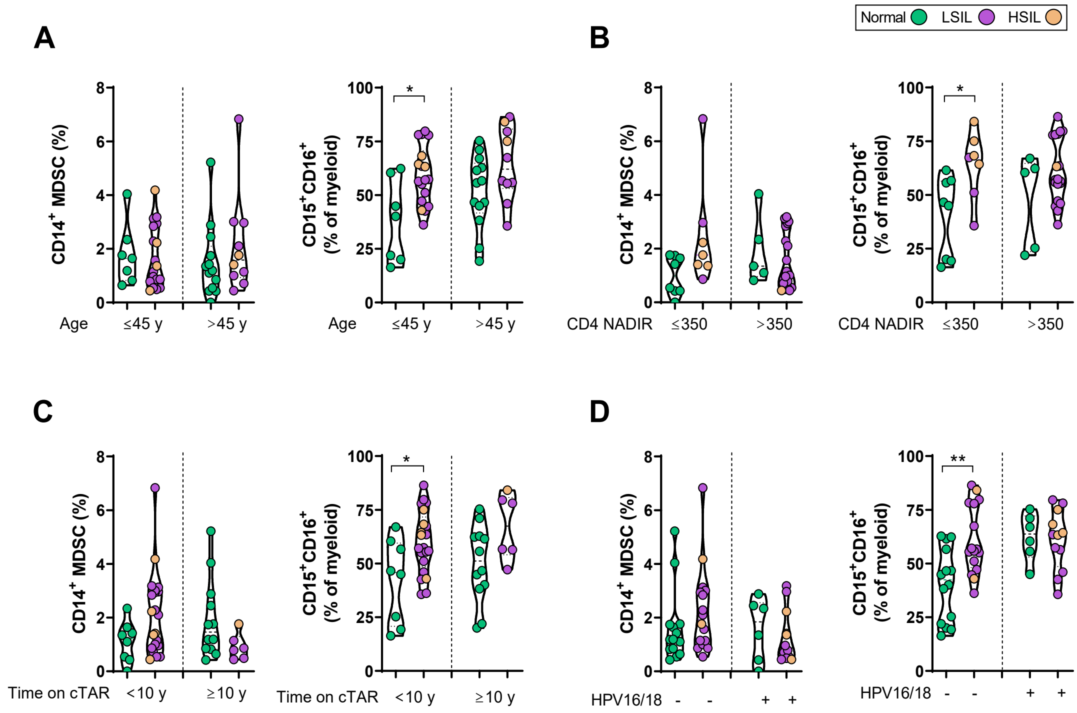

Figure S5. Differences in myeloid populations associated to squamous intraepithelial lesion by patient characteristics. Frequency of CD14$^+$ MDSC out of the CD11b^{dim}CD33$^+$ myeloid gate; frequency of CD15$^+$CD16$^+$ out of the total myeloid fraction in normal and pathological samples, including those with low-grade squamous intraepithelial lesions (LSIL) and high-grade squamous intraepithelial lesions (HSIL) by: **A**, age (≤45 or >45 years old); **B,** Nadir CD4$^+$ count (≤350 or >350 x10^9/L); **C,** Time on cART (<10 or ≥10 years of treatment); **D,** presence of HR-HPV genotypes (HPV-16 and HPV-18 negative or HPV-16 and HPV-18 positive). Data are represented as a violin plot representing normal patients (green) *versus* pathological (LSIL, in purple; HSIL highlighted in brown); horizontal lines are median and interquartile range. Statistical comparisons within groups using Mann-Whitney test are shown: *p<0.05; **p<0.01.

Supplementary Fig. 6

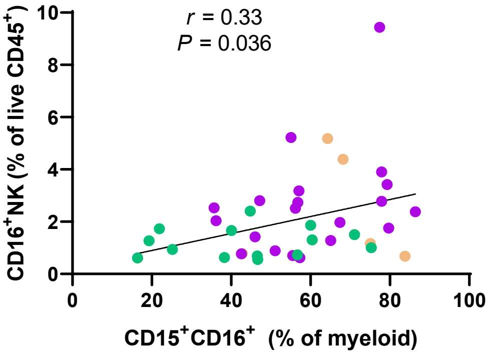

Figure S6. Correlation between the frequency of CD16^{+} NK cells and the frequency of anal CD15^{+} CD16^{+} myeloid cells in anal samples from PWH. Correlation between the frequency of CD16^{+} CD3^{-} NK cells out of the total living CD45^{+} cells, regardless of CD56 expression, and the frequency of CD15^{+}CD16^{+} out of the total myeloid fraction in normal (green), LSIL (purple) and HSIL (brown) anal samples. Statistics were performed using non-parametric Spearman rank correlation and nonlinear regression.

Supplementary Fig. 7

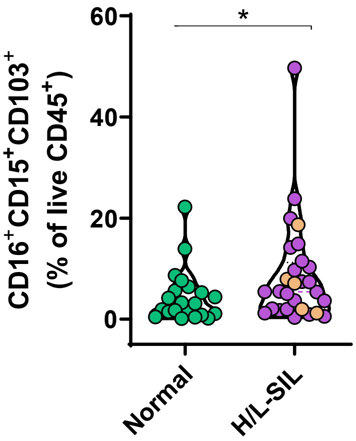

Figure S7. Frequency of CD103$^+$ neutrophils associated to squamous intraepithelial lesion. Frequency of neutrophils (CD15$^+$CD16$^+$) out of all living CD45$^+$ cells in normal (in green) *versus* pathological (LSIL, in purple; HSIL highlighted in brown) samples. Data are represented as a violin plot; horizontal lines are median and interquartile range. Statistical comparison using Mann-Whitney U test is shown: * $p<0.05$.

Supplementary Fig. 8

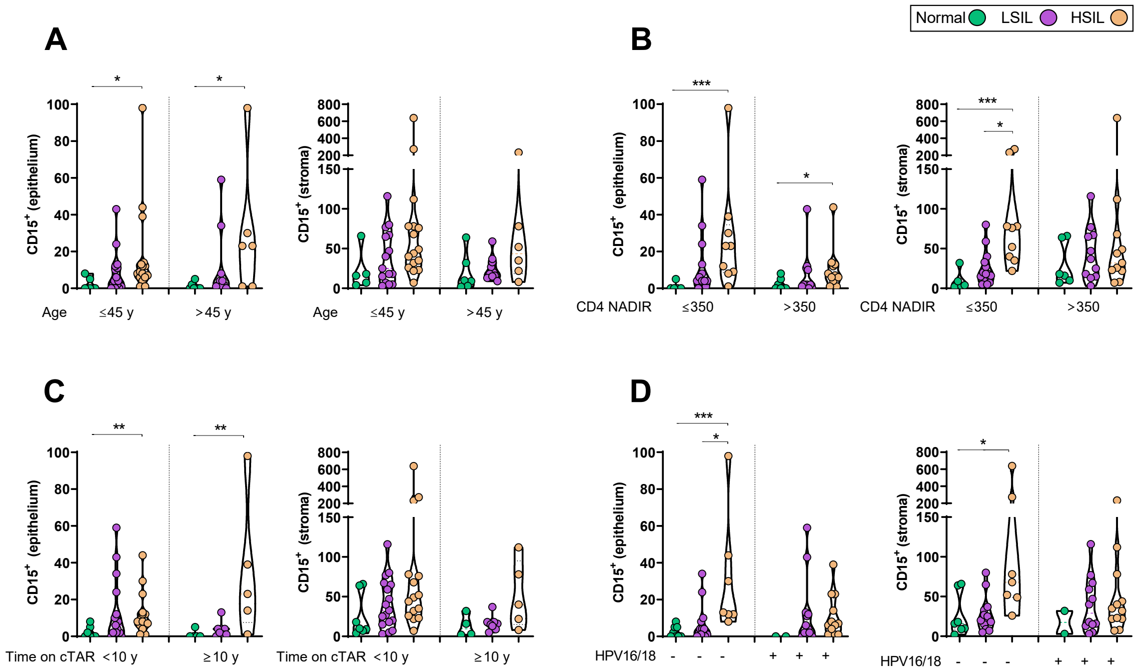

Figure S8. Differences in epithelial and stromal CD15 staining by immunohistochemistry associated to squamous intraepithelial lesion by patient characteristics. Average number of $CD15^{+}$ cells detected per a median of 3 fields (range 1 to 7) at 40x of magnification in the epithelium and lamina propia (stroma) in normal (green), LSIL (purple) and HSIL (brown) anal samples analysed by: **A**, age (≤45 or >45 years old); **B,** Nadir $CD4^{+}$ lymphocytes (≤350 or >350 $x10^{9}$/L); **C,** time on cART (<10 or ≥10 years of treatment); **D,** presence of HR-HPV genotypes (HPV-16 and HPV-18 negative or HPV-16 and HPV-18 positive). Data are represented as a violin plot; horizontal lines are median and interquartile range. Statistical comparisons within groups using non-parametric Kruskal-Wallis test with Dunn's post-hoc test for multiple comparisons are shown: *p<0.05; **p<0.01; ***p<0.001.

Supplementary Fig. 9

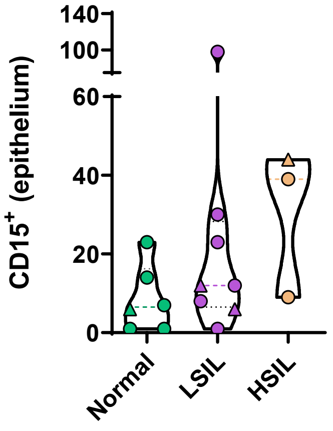

Figure S9. Predictive value of CD15^{+} quantification in the epithelium by immunohistochemistry. Average number of CD15^{+} cells detected per a median of 3 fields (range 1 to 7) at 40x of magnification in the epithelium of the HSIL group from the validation cohort by subsequent outcome within then same location: normal (in green), LSIL (in purple) or HSIL (in brown), in which triangles highlight samples with concomitant p16 negative staining. Data are represented as a violin plot; horizontal lines are median and interquartile range.

Supplementary Fig. 10

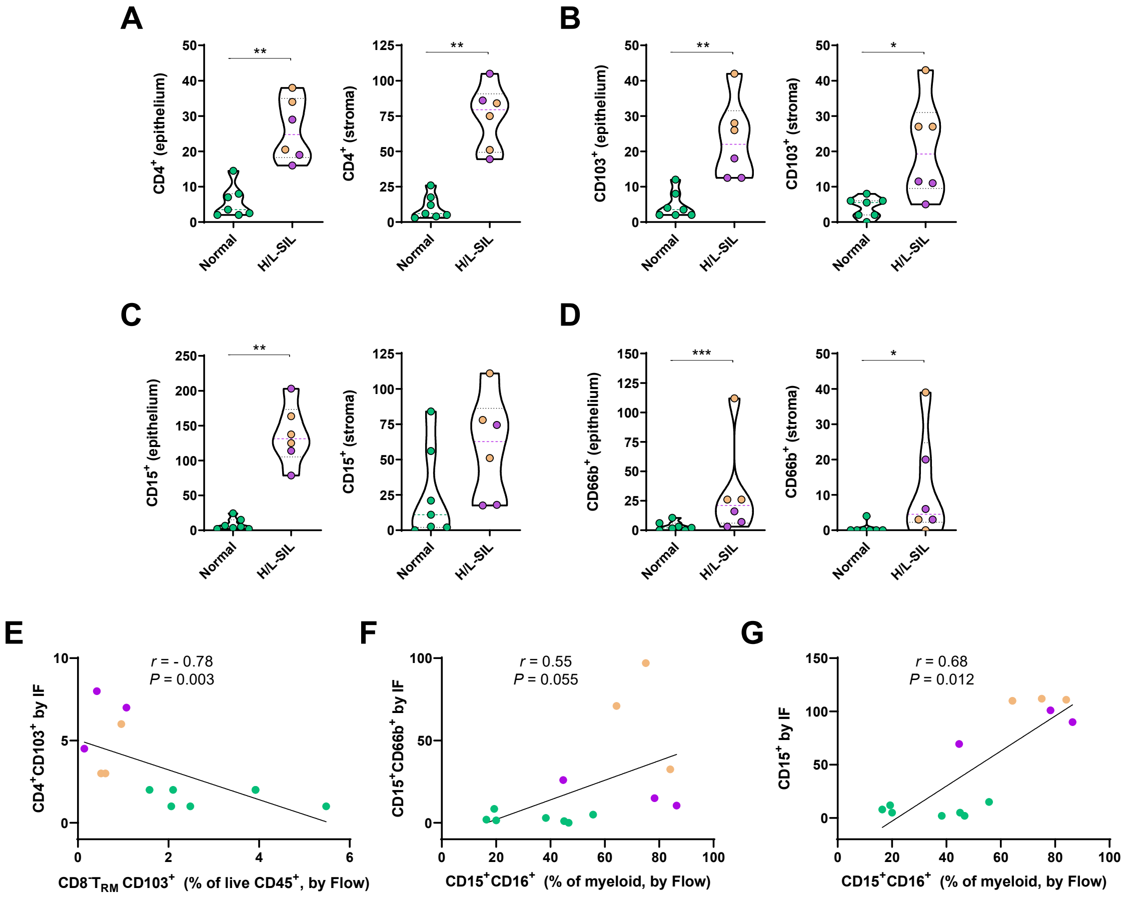

Figure S10. Comparison between marker detection by immunofluorescence and flow cytometry. A-D, Median number of single CD4 or CD103 (**A-B**) and CD15 or CD66b (**C-D**) positive cells detected per a median of 6 fields (range 2 to 15) at 25x of magnification in the epithelium or the stroma in normal (green) and pathological (LSIL, in purple; HSIL highlighted in brown) samples. Data are represented as a violin plot; horizontal lines are median and interquartile range. Statistical comparison using Mann-Whitney U test is shown: $*p<0.05$; $**p<0.01$; $***p<0.001$. **E-G,** Correlation between: (**E**) the median number of $CD4^+CD103^+$ determined by IF and the frequency of $CD103^+$ $CD8^-$ T_{RM} cells out of the total living $CD45^+$ cells determined by flow cytometry, (**F**) the median number of $CD15^+CD66b^+$ determined by IF and the frequency of $CD15^+CD16^+$ cells out of the total living myeloid cells determined by flow cytometry and, (**G**) the median number of single $CD15^+$ determined by IF and the frequency of $CD15^+CD16^+$ cells out of the total living myeloid cells determined by flow cytometry in normal (green), LSIL (purple) and HSIL (brown) paired anal biopsies of the discovery cohort. Statistics were performed using non-parametric Spearman rank correlation and nonlinear regression.
